# Supplementary material for: Prognostic and clinicopathological significance of long noncoding RNA MALAT-1 expression in patients with non-small cell lung cancer: A meta-analysis
Source: PLoS One. 2020 Oct 14;15(10):e0240321. doi: 10.1371/journal.pone.0240321 (PMC7556468; doi:10.1371/journal.pone.0240321)
Supplement: Attachment — Submitted filename: LiuXLPONE-D-20-09605_R1_Response to Reviewers.doc [file pone.0240321.s002.doc]

*Journal Requirements:*

*When submitting your revision, we need you to address these additional requirements.*

*Question1: Please ensure that your manuscript meets PLOS ONE's style requirements, including those for file naming.*

**Answer1:**

We have revised our manuscript and the charts contained in the manuscript in accordance with the submission guidelines to make it more in line with the publication requirements of PLOS ONE.

*Question2：At this time, we ask that you please provide the full search strategy and search terms for at least one database used as Supplementary Information.*

**Answer 2:**

The full search strategy and search terms are as follows:

(((((("Carcinoma, Non-Small-Cell Lung"[Mesh]) OR "Lung Neoplasms"[Mesh]) OR non-small cell lung cancer[Title/Abstract]) OR lung cancer[Title/Abstract])) AND ((((lncRNA[Title/Abstract]) OR long non-coding RNA[Title/Abstract])) OR ((((("MALAT1 long non-coding RNA, human" [Supplementary Concept]) OR NEAT2[Title/Abstract]) OR MALAT1[Title/Abstract]) OR MALAT-1[Title/Abstract]) OR Metastasis associated lung adenocarcinoma transcript 1[Title/Abstract])))

The literature search was from inception of the PubMed database to March 1, 2020, and a total of 1149 papers were retrieved using this search strategy.

*Question3：About ORCID*

**Answer 3:**

Thank you for your careful review. We will register an ORCID iD and link it to our Editorial Manager account.

*Question4：If you would like to make changes to your financial disclosure, please include your updated statement in your cover letter. Guidelines for resubmitting your figure files are available below the reviewer comments at the end of this letter.*

**Answer 4:**

This work was not financially supported by any project.

Dear Reviewer **#1**,

We thank the reviewer very much for the critical comments and constructive suggestions that have helped to greatly improve our manuscript. We have revised the manuscript according to the suggestions. Our responses to the comments are as follows:

**Reviewer #1:**

*The authors Liang et al., in their article entitled “Prognostic and Clinicopathological Significance of Long Noncoding RNA MALAT-1 Expression in Patients with Non-Small Cell Lung Cancer: A Meta-Analysis” have conducted a meta-analysis to verify the association of lncRNA MALAT1 expression with prognosis and clinicopathological features in patients with non-small cell lung cancer (NSCLC).*

*Question1. The authors have stated that “we think publication bias has little effect on the outcome”. The authors need to explain this statement.*

**Answer 1:**

According to your suggestion, we have discussed this possibility in our manuscript. Indeed, statements such as “*we think publication bias has little effect on the outcome*” are not rigorous and require further explanation and revision. The trim and fill method [1] was used to test publication bias and to identify and correct the asymmetry of the funnel graph caused by publication bias. We used Egger’s and Begg’s methods to test the publication bias. When we used Egger’s method to evaluate the LNM group data, we found that there was publication bias (*P*=0.023), so we used the trim and fill method [2, 3] to verify the publication bias. Because of the data heterogeneity, we used the random-effects model to analyze the results of the trim and fill method. The pooled results before and after trimming did not change [before trimming: (HR:1.049, 95% CI: 0.056–2.042), after trimming: (HR: 0.935 95% CI: 0.353–2.479)], so we believe that the results were stable, and the merged data were credible. （page24, 398-404）

References

[1].	Zhang T.S, Zhong W.Z. [Performance of the Nonparametric Trim and Fill Method in Stata]. [J] Evidence-Based Medicine. 2009;9(04):240-2.(Chinese)

[2].	Duval S, Tweedie R. Trim and fill: A simple funnel-plot-based method of testing and adjusting for publication bias in meta-analysis. Biometrics. 2000;56(2):455-63. doi: 10.1111/j.0006-341x.2000.00455.x. PubMed PMID: 10877304.

[3].	Peters JL, Sutton AJ, Jones DR, Abrams KR, Rushton L. Performance of the trim and fill method in the presence of publication bias and between-study heterogeneity. Stat Med. 2007;26(25):4544-62. Epub 2007/05/04. doi: 10.1002/sim.2889. PubMed PMID: 17476644.

*Question2. In the discussion section, the authors have provided information on the results obtained from previous studies. However, it will be helpful for readers if the correlation with the functional impact of MALAT1 expression on features like gender, tumor size, etc. is explained*

**Answer 2:**

We have made revised the manuscript based on your suggestion; the revisions are in red font. (page23- 24, lines 382–390；page 25, lines 408-415)

Dear Dr. Shama Prasada Kabekkodu,

Thank you very much for your comments on our manuscript entitled, “Prognostic and Clinicopathological Significance of Long Noncoding RNA MALAT-1 Expression in Patients with Non-Small Cell Lung Cancer: A Meta-Analysis” (PONE-D-20-09605). We believe that your comments will make our manuscript more acceptable.

**Reviewer #2**

*General Overview*

*The present meta-analysis was performed to investigate the association between MALAT-1 expression with prognosis in NSCLC. For this, a literature search was performed using multiple search engines. Subsequently,*

*Comments：*

1. *More key words should be used.*

*2. Manuscript should be thoroughly checked for typographical errors, grammar and English language*

*4. The authors state that “The studies were published from 2003 to 2020”. However, there are no publication in the reference quoting the studies in 2003. Hence, this sentence needs to be modifies.*

*5. The legends in the Figure S3 , S6 are very small and difficult to read. Need to provide better quality figure with increase fonts.*

**Answer 1, 2, 5:**

We have corrected these mistakes in the revised manuscript and added useful keywords. We have also carefully checked the grammar and spelling, and re-edited the picture to make it easier to read.

**Answer 4:**

The study of 2003 reference cited is listed below:

[6]	Ji P, Diederichs S, Wang W, Boing S, Metzger R, Schneider PM, et al. MALAT-1, a novel noncoding RNA, and thymosin beta4 predict metastasis and survival in early-stage non-small cell lung cancer. Oncogene. 2003;22(39):8031-41. Epub 2003/09/13. doi: 10.1038/sj.onc.1206928. PubMed PMID: 12970751.

*Question3.Authors selected studies which had used RT-PCR or ISH as inclusion. Does this also include qPCR?*

**Answer 3:**

After careful reading and inspection of the original literature, we found that all selected studies (except Schmidt et al 2011) used quantitative PCR to detect MALAT-1 expression, so we made corrections to the revised manuscript and to Table 1.

*Question6: How did the authors grade the study? A GRADE summary of the findings can be given.*

**Answer 6:**

Thank you very much for your valuable comments. To evaluate the quality of the literature, we re-evaluated the original literature and summarized the NOS into a separate table at the end of the letter, and also revised it in Table 1.

*Question7: Already a meta-analysis of MALAT-l in NSCLC is published (PMID: 31846184). Authors should describe, how their meta-analysis does is different from that published in PMID: 31846184. Further, in the limitation of the study, authors mentioned that their meta-analysis is from lung cancer tissue. Why authors did did not include the serological studies in their meta-analysis? A strong and valid justification should be provided. Why authors did not mention the same in inclusion and exclusion criteria section. Authors can perform reanalysis of the data by including the studies published in PMID: 31846184 and present the data to avoid the possible publication bias.*

**Answer 7:**

Thank you very much for your pertinent comments. We found PMID:31846184 article for Zheng *et al*. “Long noncoding RNA MALAT1 as a candidate serological biomarker for the diagnosis of non-small cell lung cancer: A meta-analysis.” Comparing the differences between the two articles, we think that study（PMID: 31846184）entitled, “Diagnostic studies relevant to circulation long noncoding RNA MALAT1 as a candidate serological biomarker for NSCLC,” which used meta-analysis of diagnostic tests, including six studies with eight datasets, analysis of circulating blood lncRNA MALAT-1 expression sensitivity, specificity, positive likelihood ratio, negative likelihood ratio and diagnostic odds ratio, suggest that it has a high diagnostic value for NSCLC and a low misdiagnosis rate. However, our study takes into account the correlation of high expression of lncRNA MALAT-1 and the prognosis of patients with NSCLC. It also used meta-analysis of survival data to analyze the relationship between the expression of lncRNA MALAT-1 and the prognostic survival of patients with NSCLC. We think the focus of these article are different, serum long noncoding RNA MALAT1 is a promising biomarker for NSCLC screening. In our study, tissue samples for MALAT-1 were mainly aimed at the prognosis assessment of NSCLC. In addition, in order to reduce statistical heterogeneity and make the results more stable, credible, and more convincing, we only included lung tissue specimens as the research object, tissue sample can make the meta-analysis results more Reliable and more clinically convincing. At the same time, in the suggestions you give us, we also found that the exclusion criteria and the Discussion section were not rigorous. We have revised the manuscript and marked the revisions in red font (page 7, lines 127–128; page 26, lines 431-440).

Table Methodological Quality of Studies Included in the Final Analysis Based on the Newcastle-Ottawa-Scale for Assessing the Quality of Case-Control Studies

| **Studies** | **Selection(score)** | | | | | **Comparability(score)** | **Exposure(score)** | | | **Total scores** |
| --- | --- | --- | --- | --- | --- | --- | --- | --- | --- | --- |
|  | the case definition adequate | Representativeness  of the cases | | Selection of Controls | Definition of Controls | Basis on the  design or analysis | Ascertainment  of exposure | Same methods of ascertainment | Non-Response rate |  |
| Jin et al.(2020)^25^ | 1 | 1 | 0 | | 0 | 1 | 1 | 1 | 1 | 6 |
| Peng et al.(2017)^18^ | 1 | 1 | 0 | | 0 | 2 | 1 | 1 | 1 | 7 |
| Zhang et al.(2016)^29^ | 1 | 1 | 0 | | 0 | 2 | 1 | 1 | 1 | 7 |
| Zhang et al.(2015)^30^ | 1 | 1 | 0 | | 0 | 1 | 1 | 1 | 1 | 6 |
| Mu et al.(2013)^14^ | 1 | 1 | 0 | | 0 | 1 | 1 | 1 | 1 | 6 |
| Ma et al.(2013)^31^ | 1 | 1 | 0 | | 0 | 1 | 1 | 1 | 1 | 6 |
| Yang et al.(2019)^26^ | 1 | 1 | 0 | | 0 | 1 | 1 | 1 | 1 | 6 |
| Xiao et al.(2019)^27^ | 1 | 1 | 0 | | 0 | 1 | 1 | 1 | 1 | 6 |
| Lin et al.(2018)^15^ | 1 | 1 | 0 | | 0 | 1 | 1 | 1 | 1 | 6 |
| Tang et al.(2018)^17^ | 1 | 1 | 0 | | 0 | 1 | 1 | 1 | 1 | 6 |
| Jen et al.(2017)^12^ | 1 | 1 | 0 | | 0 | 1 | 1 | 1 | 1 | 6 |
| Schmidt et al.(2011)^13^ | 1 | 1 | 0 | | 0 | 2 | 1 | 1 | 1 | 7 |
| Ji et al.(2003)^6^ | 1 | 1 | 0 | | 0 | 1 | 1 | 1 | 1 | 6 |
| Wang et al.(2018)^28^ | 1 | 1 | 0 | | 0 | 1 | 1 | 1 | 1 | 6 |
| Chen et al.(2017)^16^ | 1 | 1 | 0 | | 0 | 2 | 1 | 1 | 1 | 7 |
|  |  |  |  | |  |  |  |  |  |  |
